# A comparison of cardiac motion analysis software packages: application to left ventricular deformation analysis in healthy subjects

**DOI:** 10.1186/1532-429X-18-S1-P47

**Published:** 2016-01-27

**Authors:** Haifa M Almutairi, Mohammed Y Khanji, Redha Boubertakh, Marc E Miquel, Steffen E Petersen

**Affiliations:** 1 Centre for Advanced Cardiovascular Imaging and Research, William Harvey Research Institute, Queen Mary University London, London, UK; 2 Barts Health NHS Trust, London, UK

## Background

Feature tracking (FT) software packages measure myocardial wall motion deformation parameters through the cardiac cycle. Myocardial tagging technique is currently considered the gold standard for myocardial deformation measurements. This study compares 2 FT-software packages with a tagging software package and investigates the differences in strain deformation parameters measured in healthy subjects.

## Methods

41 healthy subjects were prospectively enrolled to undergo CMR examinations; one was excluded for poor image quality. CMR images were acquired using a 1.5T Achieva Philips scanner (Best, the Netherlands) and a dedicated 32-channel cardiac coil. Balanced-SSFP breath hold cine-images were acquired in the following planes: short axis (basal, mid, apical levels), 2-chamber and 4-chamber. Tagged images, (3 short axis slices of the LV (base, mid and apex), 4-chamber and 2-chamber planes) were acquired using CSPAMM, with a tag separation of 7.5 mm and a tag grid angle of 90°.

Endocardial and epicardial borders of LV were manually delineated at the end diastolic phase. Quantitative deformation parameters: strains were calculated semi-automatically using the following software: 2D Cardiac Performance Analysis, MR (TomTec Imaging Systems, Munich, Germany) and CVI42 (Circle Cardiovascular Imaging Inc. Calgary, Canada). Tagged images were analyzed using CIMTag2D software (CIMTag2D v.8.1.2, Auckland MRI Research Group, New Zealand). All statistical analysis was carried out using SPSS (IBM Corporation, Armonk, New York, USA).

## Results

Results of global circumferential, radial, and longitudinal strain means are given in Table 1

**Table 1:** Global strain measurements for the different software (mean ± standard deviation %), grey cells showed there was a significant difference (p < 0.05) between that results and CIMTag measurements.

		Tagging	Feature Tracking	Feature Tracking
Parameters	Parameters	CIMTag	CVI42	Tomtec
Circumferential Strain	SAX-basal	-17.55 ± 1.40	-16.36 ± 3.87	-15.63 ± 3.26
Circumferential Strain	SAX-mid	-18.26 ± 3.63	-14.11 ± 1.95	-14.35 ± 2.64
Circumferential Strain	SAX-apical	-20.65 ± 2.79	-14.75 ± 2.59	-17.47 ± 4.29
Radial Strain	SAX-basal	38.94 ± 26.74	29.75 ± 19.61	34.39 ± 10.68
Radial Strain	SAX-mid	27.81 ± 9.95	21.67 ± 4.15	38.55 ± 11.76
Radial Strain	SAX-apical	23.09 ± 10.52	24.67 ± 5.59	30.10 ± 13.41
Radial Strain	2-chamber	24.77 ± 12.58	30.72 ± 6.05	34.00 ± 10.96
Radial Strain	4-chamber	20.44 ± 15.46	35.15 ± 7.07	28.88 ± 7.21
Longitudinal Strain	2-chamber	-14.25 ± 2.33	-16.27 ± 2.05	-15.83 ± 3.88
Longitudinal Strain	4-chamber	-14.56 ± 2.20	-17.03 ± 1.97	-16.49 ± 3.74

There were significant differences (P < 0.05) in circumferential (basal, mid, apical) strains measured by Tomtec compared to CIMTag measurements. The differences were also statistically significant for circumferential (mid, apical) strains measured by CVI42 compared to CIMTag measurements.

Longitudinal strains measured by CVI42 compared to CIMTag showed significant differences, and most parameters measured by all FT-software packages were higher than CIMTag in absolute values. However, longitudinal strains measured by Tomtec compared to CVI42 showed no significant difference.

## Conclusions

From our results, FT- software packages measurements showed significant differences in most parameters when compared to the tagging results (CIMTag) with only a few parameters in agreement. There is a need for a standard method of validation, ideally based on a numerical phantom to assess the accuracy of these software packages in order to facilitate their use in a clinical setting.

**Figure 1:**
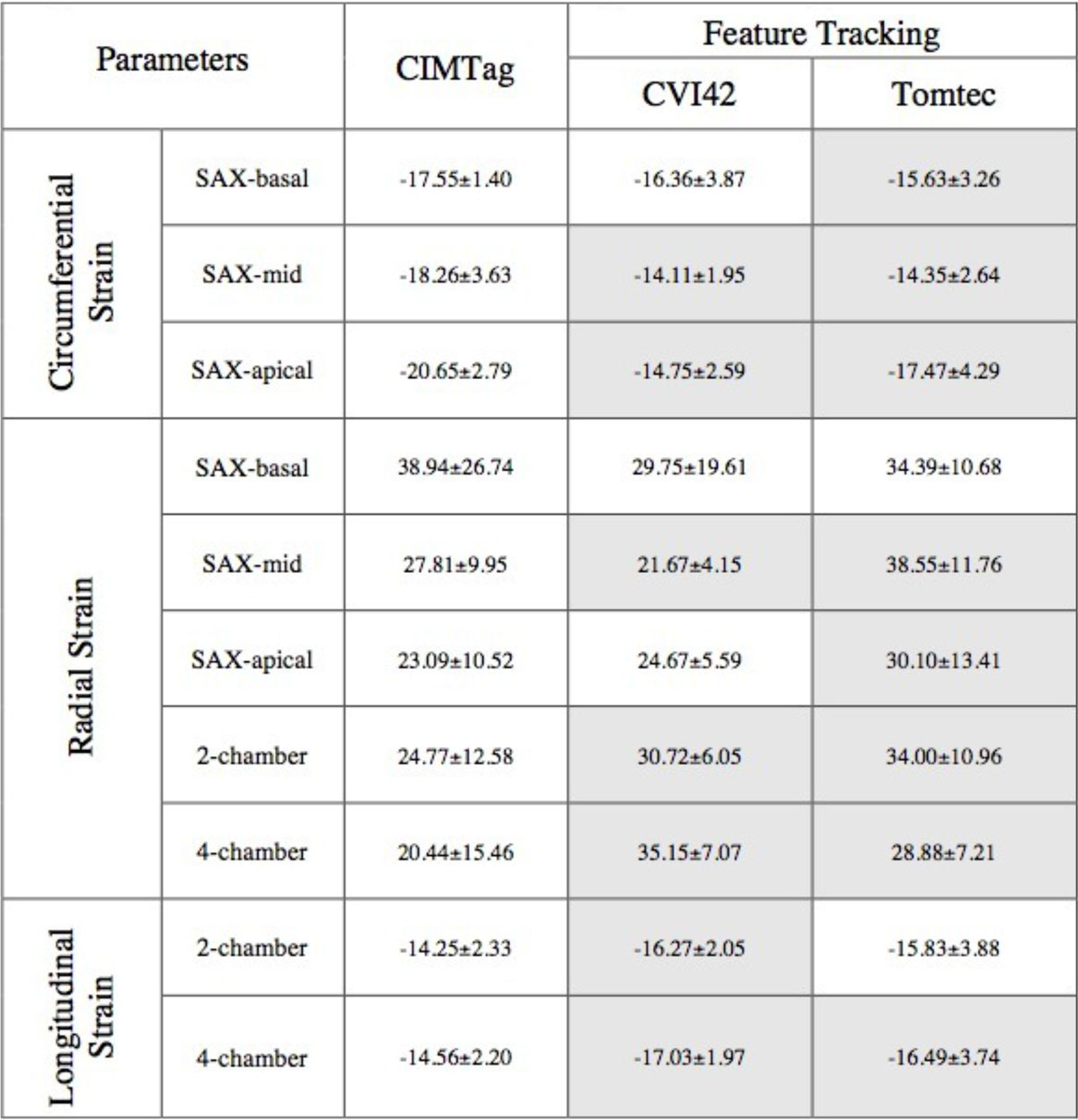
Figure 1

